# Matching Cases and Controls Using SAS® Software

**DOI:** 10.3389/fdata.2019.00004

**Published:** 2019-05-08

**Authors:** Laura Quitzau Mortensen, Kristoffer Andresen, Jakob Burcharth, Hans-Christian Pommergaard, Jacob Rosenberg

**Affiliations:** ^1^Center for Perioperative Optimization, Department of Surgery, Herlev Hospital, Herlev, Denmark; ^2^Department of Surgery, University Hospital of Zealand, Koege, Denmark; ^3^Department of Surgery and Transplantation, Rigshospitalet, Copenhagen, Denmark

**Keywords:** SAS® software, matching, code, macro, case control study, observational study, explanation, code:sas

## Abstract

Matching is frequently used in observational studies, especially in medical research. However, only a small number of articles with matching programs for the SAS software (SAS Institute Inc., Cary, NC, USA) are available, even less are usable for inexperienced users of SAS software. This article presents a matching program for the SAS software and links to an online repository for examples and test data. The program enables matching on several variables and includes in-depth explanation of the expressions used and how to customize the program. The selection of controls is randomized and automated, minimizing the risk of selection bias. Also, the program provides means for the researcher to test for incomplete matching.

## Description and Application

Matching of one group with specific exposures or outcome with a comparable group is frequently used in medical research to investigate the association between exposure and outcome. In case-control studies the cases with the disease of interest are matched with disease-free controls in order to retrospectively explore risk-factors. In matched cohort studies, the persons with the exposure of interest are matched with unexposed persons in order to make the two groups more similar with regard to confounding variables. Therefore, matching often reduces the risk of confounding in cohort studies (Bloom et al., 2007). For researchers new to the SAS software (SAS Institute Inc., Cary, NC, USA), matching cases with controls may not be easily performed. Only a small number of suitable methods for matching case with controls are available using SAS software for inexperienced SAS-users. One article described a matching program (Mounib and Satchi, 2000); however, for inexperienced SAS-users several issues hindered meaningful use of this method. These issues included typographical errors that prevented the execution of the code.

This article presents a revised matching program which was generated using SAS software, Version 9.4 of the SAS System. The program has also been tested and found applicable for SAS Software University Edition. The purpose of the code is to enable matching between two dissimilar groups based on specific characteristics selected by the user. In this article, the main focus for the use of the code is case-control matching. The revised program makes it possible to match on several variables, both on fixed values and ranges, and includes a more in-depth explanation of the expressions used. It provides a random selection of eligible controls and clarifies which cases, if any, that do not receive the desired number of controls. Thus, the aim of this paper is to present an easily understood and easy-to-use matching program for the SAS software.

## Methods

Before matching, it is necessary to prepare a data set containing all cases as well as potential controls. In this paper, this data set is referred to as *population*. One way to distinguish the two groups is to create a variable, called *casecontrol*, which is marked 1 for cases and 0 for potential controls. Since the following program matches controls to cases by age and sex, *population* must contain this information, as well as a unique identifier for each person in the data set. The program will be presented and explained from beginning to end using the lines of code as a reference. The program is presented in Tables 1–**5**. To access the program online, please go to: https://github.com/lauraqmortensen/sas.

**Table 1:** Lines 1–2.

**Line**	**Code**
1.	%LET agerange = 5;
2.	%LET ratio = 3;

*In these lines, the accepted age range between a case and a control and the desired number of controls per case are defined. The age range must be defined by the same measurement as age, i.e., years, months*.

## Lines 1–10

The program matches controls to cases by a numerical range and a categorical value, age, and sex, respectively. In *lines 1–2*, the variables *agerange* and *ratio* are defined by a *%LET* statement as macro-variables and can be recalled later with a preceding ampersand, see Table 1. The %*LET* statement in the beginning of the code makes it easy to customize the variables. The value of *agerange* has been set to five, which will allow the program to select controls of up to 5 years younger or 5 years older than the case, given that the variable *age* is listed in years. The *ratio* has been defined as three which means that to each case three unique controls will be matched. It is possible to exchange these two variables for others or match on more than two variables. However, narrowing ranges and increasing numbers of matching variables will limit the number of available controls.

**Figure 1:**
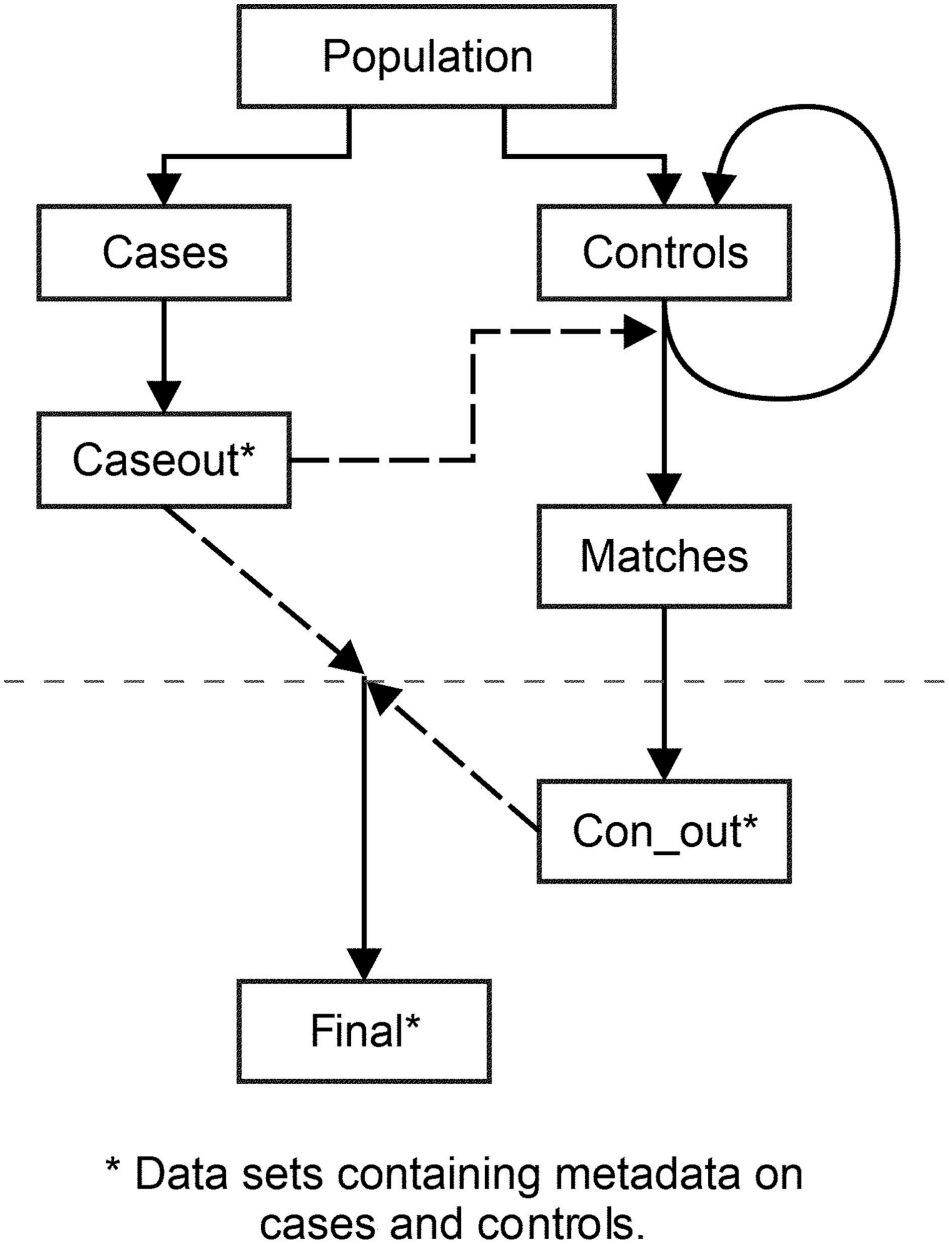
The assigning of controls to cases. Illustration of data sets used and produced by the program. The data set *controls* holds all potential controls. Controls are matched to cases based on the data set *caseout*. Matched controls are moved to *matches*; the remaining controls are reused as potential controls in *controls*. The dataset *final* contains metadata on cases and matched controls and is used to check for insufficient matches.

The purpose of *lines 3–7* is to produce two separate data sets from *population*, see Table 2 and Figure 1. One data set contains cases (*cases)*; the other holds potential controls (*controls)*. The separation of cases and possible controls is done by the variable *casecontrol* in the data set containing the entire population, *population*.

**Table 2:** Lines 3–7.

**Line**	**Code**
3.	DATA cases controls;
4.	SET population;
5.	IF casecontrol = 1 THEN OUTPUT cases;
6.	ELSE OUTPUT controls;
7.	RUN;

*Population is a data set containing both cases and possible controls. This data set should have a variable called “casecontrol” and a variable called “uniqueid.” The data set should also include the matching variables. The data step creates two new data sets, cases and controls*.

*Lines 8–10* produce a data set, *caseout*, containing frequencies of different combinations in the case group of the matching variables, *age* and *gender*, see Table 3. This list will later be used to identify eligible controls. The *NOPRINT* option suppresses display of an output from the *PROC FREQ*.

**Table 3:** Lines 8–10.

**Line**	**Code**
8.	PROC FREQ NOPRINT DATA = cases;
9.	TABLES age^*^gender/OUT = caseout;
10.	RUN;

*A new data set caseout is created. It holds count of various combinations of matching variables within the case group*.

## The Macro, Lines 11–34

A macro is a program within the program. It allows for fast changes and is helpful and time-saving when the same code needs to run many times as is done when matching controls to cases (Slaughter and Delwiche, 2004). The macro, named *sample*, is repeated for each combination of matching variables in *caseout* to locate eligible controls for matching, see Table 4.

**Table 4:** The macro, lines 11–38.

**Line**	**Code**
11.	%MACRO sample(v_age, v_gender, v_count);
12.	DATA qualify1;
13.	SET controls;
14.	WHERE (&v_age-&agerange <= age <= &v_age+&agerange)
	AND
	(gender = “&v_gender”);
15.	case_age = &v_age;
16.	case_gender = “&v_gender”;
17.	SEED = RANUNI(0);
18.	PROC SORT;
19.	BY SEED;
20.	DATA qualify2;
21.	SET qualify1 NOBS = totobs;
22.	IF _N_ <= &v_count^*^&ratio;
23.	IF &v_count^*^&ratio <= totobs THEN tag = ‘yes’;
24.	ELSE tag = ‘no’;
25.	PROC APPEND BASE = matches DATA = qualify2 force;
26.	PROC SORT DATA = qualify2 OUT = temp1 (KEEP = uniqueid);
27.	BY uniqueid;
28.	PROC SORT DATA = controls OUT = temp2;
29.	BY uniqueid;
30.	DATA controls;
31.	MERGE temp1(IN = in1) temp2(IN = in2);
32.	BY uniqueid;
33.	IF in2 AND NOT in1;
34.	%MEND sample;
35.	DATA _NULL_;
36.	SET caseout;
37.	CALL EXECUTE
	(‘%sample(‘||age||’,‘||gender||’,‘||count||’)’);
38.	RUN;

*The macro is a program within the program. The macro begins with the %macro statement and ends with the %mend statement. It is started with the call execute statement. The macro runs in iterations for each combination of matching variables in the case group. The matched controls are placed in the new data set, matches*.

In *line 11*, the macro is begun with the *%MACRO* statement. Parameters are introduced to the macro definition in a set of parentheses. These parameters are called within the macro with a preceding ampersand. The parameters will be defined later when the macro is invoked.

*Lines 12–14* produce a data set, *qualify1*, which contains eligible controls based on the matching criteria set forth in *line 14*. That is, in order to be an eligible control, the following must apply; the value of the variable *age* must be equal to or greater than the value of *age* of the case minus the value of *agerange* and be less than or equal to the value of *age* of the case plus the value of *agerange*. Furthermore, the gender of the potential control must equal that of the case. The variable *v_gender* is in quotation marks because it is a categorical variable. New variables are introduced to *qualify1* in *lines 15–16* for the controls, representing values of gender and age of their corresponding case.

The new variable, *SEED*, introduced in *lines 17–19* is assigned a random number for each eligible control in the data set. This random number has a value between 0 and 1 and is produced from the *RANUNI* function. The *RANUNI* distributes the numbers in a uniform manner. The *(0)* instructs the SAS software to start the sequence of numbers based on the time of day which makes it impossible to replicate the stream of random numbers. The variable *SEED* is renewed with each iteration of the macro. Therefore, the potential controls who have not been assigned to a case, reenter the group of potential controls in *controls* and receive a new random number with the new iteration of the macro, see Figure 1.

The purpose of *lines 20–24* is to output a data set, *qualify2*, which will contain the required number of controls, if accessible; otherwise, it will contain the maximal obtainable number. Several variables are introduced in these five lines. The value of the variable *NOBS* equals the total number of potential controls in *qualify1*. With the statement *NOBS* = *totobs*, the variable *totobs* is assigned the value of *NOBS. Totobs* can be used anywhere in the data step. The variable *_N_* is an automatic variable in the SAS software which is 1 for the first observation of the data set and increases by 1 for each additional observation. *V_count* represents the variable *count* in the data set *caseout* which keeps count of the frequency of combinations of matching variables. The consequence of *line 22* is that *qualify2* will contain the assigned controls. If *qualify1* holds fewer observations than the number of desired controls, then *qualify2* will obtain them all. However, if *qualify1* has more observations than required, *qualify2* will obtain the first observations until the observation number, _N_, equals the number of desired controls, &v_count^*^&*ratio*. In effect, the macro will not assign too many controls. Since the potential controls have been sorted by *SEED*, those with the smallest values in *SEED* will be acquired. The variable *tag* is designated “yes” or “no” based on whether or not the desired amount of controls is reached.

In *line 25* the matched controls from *qualify2* are added to the end of the data set *matches* using the *APPEND* procedure. *Matches* will contain all the final controls and will not exist until the first iteration of the macro. The two data sets, *qualify2* and *controls*, are sorted in *lines 26–29* by *uniqueid*, an identifier unique for each participant. The outcome is two new data sets, *temp1* and *temp2*. *Temp1* contains the newly found controls that were added to *matches* in *line 25*, and *temp2* contains the original pool of potential controls in *controls* that *qualify1* was created from and form which *qualify1* will be created again during next iteration of the macro.

The purpose of *lines 30–33* is to delete the already matched controls from the data set *controls* in order to ensure that controls are used only once. The *IN* = option creates temporary variables, *in1* and *in2*. In *line 33* these two variables are used for selecting only non-matched controls for the updated data set *controls*. The macro is ended with the *%MEND* statement in *line 34*.

## Lines 35–38

The purpose of these lines is to execute the macro, see Table 4. In *line 35* the data statement is followed by the word _*NULL*_ which is a keyword that prevents the SAS software from writing a new data set (SAS Institute Inc, 2001). Execution of the macro is implemented with a *CALL EXECUTE* statement. The macro is run on the basis of the *caseout* data set, and the macro parameters are defined as the variables *age, gender*, and *count* from *caseout*. The double vertical bar around the variables links data from the data step to the macro (Usov, 2014). Because of the preceding *SET* statement, the *CALL EXECUTE* executes the macro repeatedly for each observation in the *caseout* data set.

The result of the macro is two data sets. First, the new data set *matches* which contains the controls that have been selected. Second, an updated version of the data set *controls* which contains the rest of the population that has not been selected as controls.

## Lines 39–54

This section is run in order to discover which, if any, cases were not matched with a sufficient number of controls.

The outcome of *lines 39–40* is the data set *con_out*, the equivalent of *caseout* for controls, see Table 5. *Con_out* contains frequencies of controls for each unique combination of matching variables. In *lines 41–44*, the data set *con_out* and the data set *caseout* are sorted by the same variables. The two data sets are merged in *lines 45–47*, resulting in the data set *final* which contains numbers of cases with each unique combination of matching variables, as well as the number of matched controls for each combination.

**Table 5:** Lines 39–54.

**Line**	**Code**
39.	PROC FREQ NOPRINT DATA = matches;
40.	TABLES case_age^*^case_gender/OUT = con_out;
41.	PROC SORT DATA = caseout(RENAME =
	(age = case_age gender = case_gender
	count = case_cnt));
42.	BY case_age case_gender;
43.	PROC SORT DATA = con_out (RENAME = (count = con_cnt));
44.	BY case_age case_gender;
45.	DATA final (DROP = percent);
46.	MERGE caseout con_out;
47.	BY case_age case_gender;
48.	con_need = case_cnt^*^&ratio;
49.	IF con_cnt = . THEN con_cnt = 0;
50.	diff = con_cnt-con_need;
51.	PROC PRINT DATA = final;
52.	WHERE diff< 0;
53.	TITLE ‘Insufficient Matches’;
54.	RUN;

*To investigate whether all cases have received the desired amount of matched controls, the code in this table may be used. It results in a table showing the cases missing one or more matched controls*.

Two new variables are created in *lines 48–50, con_need* and *diff* . They represent the number of controls needed for each type of case and the difference between the number of controls matched and needed, respectively. Finally, in *lines 51–54*, the table named “insufficient matches” is created from the data set *final*. The table contains the cases who did not receive a sufficient number of matched controls.

## Discussion

The program presented in this paper uses individual matching, i.e., matching of controls to cases based on specific characteristics. The program makes it possible to match on several variables. It provides a random selection of eligible controls and ensures that each control is only used once. In the event of more than one control per case, the program will assign all controls before moving on to the next case. This strategy is timesaving and useful with large data sets. The program has been revised from its previous edition (Mounib and Satchi, 2000) which contained several issues that impeded its use. In its current form, the program has successfully been used by the authors to match multiple controls to more than 40,000 cases (Mortensen et al., 2017).

The program is easy to customize. In order to widen or narrow the age span or match more or fewer controls it is possible to change the value of *agerange* and/or *ratio*. Often, it will not be necessary to match on other variables than age and gender. However, it is possible to add matching variables to the program. For instance, in order to match on ethnicity, a categorical variable that describes ethnic background, *ethnic*, is needed in the *population* data set. The changes that need to be made in the program are listed in bold in Table 6.

**Table 6:** An example of addition of a matching variable.

**Line**	**Code**
9.	TABLES age^*^gender^*^ethnic/OUT = caseout;
11.	%MACRO sample(v_age, v_gender, v_count,**v_ethnic**);
14.	WHERE (&v_age-&agerange <= age <= &v_age+&agerange)
	AND
	(gender = “&v_gender”)
	**AND**
	(ethnic**=** “&v_ethnic”);
16.	case_gender = “&v_gender”;
	**case_ethnic**=** “&v_ethnic”;**
37.	CALL EXECUTE
	(‘%sample(‘||age||’,‘||gender||’,‘||count||’
	**,‘||ethnic||’)');**
40.	TABLES case_age^*^case_gender^*^case_ethnic/OUT = con_out;
41.	PROC SORT DATA = caseout(RENAME =
	(age = case_age gender = case_gender count = case_cnt
	** ethnic**=**case_ethnic));**
42.	BY case_age case_gender **case_ethnic;**
44.	BY case_age case_gender **case_ethnic;**
47.	BY case_age case_gender **case_ethnic;**

*The table contains the lines of the program that need changing at the addition of an extra matching variable (ethnic). The lines are numbered corresponding to the lines in Tables 1–5, and the changes are written in bold*.

When having a small number of possible controls and/or very strict matching criteria, the program presented here may fall short, and another strategy may be considered, where the program locates controls for the cases with fewest possible controls first (Wang, 2012). However, this option will be time-consuming for larger data sets. It has not been possible to test the code in other versions of the SAS Software than Version 9.4 and University Edition due to inaccessibility. It is therefore not possible for us to guarantee the applicability to other versions.

## Conclusion

We have presented a program for randomized matching of controls to cases using SAS software. Variables, statements, and options have been explained to ease understanding of the code for researchers less used to working with the software. The program can handle several matching variables and an adjustable ratio of controls. The selection of controls is randomized and automated which minimizes the risk of selection bias. Also, the program produces a variable as well as a table by which the researchers can examine which cases did not receive the desired number of controls.

SAS and all other SAS Institute Inc. product or service names are registered trademarks or trademarks of SAS Institute Inc. in the USA and other countries. ®indicates USA registration.

## Author Contributions

All authors contributed to the conception of the work. LM drafted the manuscript. KA, JB, H-CP, and JR revised the manuscript critically. All authors have given final approval and are accountable for the content of this article.

### Conflict of Interest Statement

The authors declare that the research was conducted in the absence of any commercial or financial relationships that could be construed as a potential conflict of interest.

## References

[B1] Bloom M. S. Schisterman E. F. Hediger M. L. (2007). The use and misuse of matching in case-control studies: the example of polycystic ovary syndrome. Fertil. Steril. 88, 707–710. 10.1016/j.fertnstert.2006.11.125 17433314 PMC2040105

[B2] Mortensen L. Q. Burcharth J. Andresen K. Pommergaard H. C. Rosenberg J. (2017). An 18-year nationwide cohort study on the association between diverticulitis and colon cancer. Ann. Surg. 265, 954–959. 10.1097/SLA.0000000000001794 27192351

[B3] Mounib E. L. Satchi T. (2000). Automating the selection of controls in case-control studies, in Proceedings of the Twenty-Fifth Annual SAS® Users Group International Conference (Indianapolis, IN).

[B4] SAS Institute Inc (2001). Writing lines to the SAS log or to an output file, in Step-by-Step Programming with Base SAS® *Software* (Cary, NC: SAS Institute Inc.), 521–536.

[B5] Slaughter S. J. Delwiche L. D. (2004). SAS® macro programming for beginners, in Proceedings of the Twenty-Ninth Annual SAS® Users Group International Conference (Montreal, QC).

[B6] Usov A. (2014). Call execute: let your program run your macro, in Conference Paper Presented at Pharmaceutical Users Software Exchange Annual Conference (London, UK).

[B7] Wang Z. (2012). Optimized 1:N case-control match using SAS®, in Proceedings of the SAS® Global Forum 2012 Conference (Orlando, FL).

